# Recognition of American Veterinarians

**Published:** 1890-04

**Authors:** 


### EDITORIAL.

### RECOGNITION OF AMERICAN VETERINARIANS.

We have on record but two Americans of note in other professions than our own who have attempted to aid Veterinary Medicine, until within a very few years. Dr. Benjamin Rush, of Philadelphia, stimulated by the success of the recently established Veterinary Schools of Europe, in 1806 attempted, and failed to awaken, any active interest among the tranquil agriculturists who surrounded him ; and Dr. S. D. Gross, the great surgeon, in 1870 offered to the American Medical Association a vigorous set of resolutions and an urgent appeal for the advancement of veterinary studies, which was met with equally narrow-minded refusal. The earliest U. S. Veterinary School (1852) died in embryo. The New York College of Veterinary Surgeons, which commenced teaching in 1861 ; its offspring, the American Veterinary College, which had birth in 1875, and later, schools in Boston, Philadelphia, Chicago, Minneapolis and Baltimore, have all met with varied success. Only one has met with any suspicion of liberality from the community in which it originated. The General government, State governments and rich agriculturists and educators have been appealed to for help in advancing the profession which furnishes physicians for the health of the 165,285,363 animals constituting $2,418,766,028 of the wealth of the United States in vain.

The 12,000 animals of the U. S. Army, which cost the government $2,200,000, have been examined for purchase, condemned for disease, regulated in their food and shoeing, by officers whose scientific training is in strategy, grand tactics, logistics, engineering, marches, battles and the use of fire-arms, and whose experience with horses is as variable as that of the empirics who for years made up the only veterinary body of the country. Only within a few years in the United States have the teachers and students of human medicine and the public conceded that Veterinary Medicine held a position equally scientific with the other professions, and that its advancement would be to the material and economical advantage of the country.

This recognition has been forced from them by the personal struggles of those veterinarians themselves who have made success of the schools in which they taught, and who have leavened the prejudices of the public by sprinkling their intelligent graduates into the Department of Agriculture, the boards of State commissions, the laboratories of the universities, and into the horse and cattle stables of the great cities and richer agricultural communities.

It is an old truth that a "prophet is not without honor, save in his own country and in his own house."

We congratulate Professor A. Liautard that his work in the American Veterinary College, with its 350 graduates in fifteen years, has at last obtained the honor of government recognition; the FRENCH GOVERNMENT, by a decree of the Minister of Agriculture, has ordered that the holders of the diplomas given by the American Veterinary College may be, by virtue of this diploma, authorized to compete for the French diploma after having passed in one of their schools the time assigned to them, according to their degree of instruction, by the council of professors in the school in which they wish to follow their course.

In face of the previous non-recognition of our veterinary schools by those in Europe, and coming immediately after the decree of the University of Berlin refusing to recognize any American medical diplomas, this grant is of exceptional importance.

